# High Genetic Diversity among *Stenotrophomonas maltophilia* Isolates from Single Hospital: Nosocomial Outbreaks or Genotypic Profile Changes during Subcultures

**DOI:** 10.21315/mjms2018.25.2.5

**Published:** 2018-04-27

**Authors:** Meryem Güvenir, Baris Otlu, Emine Tunc, Elif Aktas, Kaya Suer

**Affiliations:** 1 Near East University, Vocational School of Health Services, Nicosia, Cyprus; 2 Inonu University Department of Medical Microbiology, Faculty of Medicine, Malatya, Turkey; 3 Near East University, Department of Clinical Microbiology and Infections Diseases, Nicosia, Cyprus; 4 Sisli Etfal Research and Training Hospital, Department, of Clinical Microbiology, Istanbul, Turkey

**Keywords:** Stenotrophomonas maltophilia, multiclonal outbreaks, hypermutation

## Abstract

**Background:**

*Stenotrophomonas maltophilia* is a non-fermentative gram-negative bacillus which is widely recognised as an important nosocomial pathogen causing pneumonia, blood-stream, wound and urinary tract infections, particularly in immunosuppressed patients. The aim of this study was to evaluate a nosocomial outbreak of by *S. maltophilia* in an intensive care unit of a tertiary hospital and evaluate unexpected multiclonality.

**Methods:**

A total of 11 isolates from respiratory cultures in intensive care unit of a 24 bed tertiary hospital obtained over a one months period and one isolate obtained from the nebuliser during environmental screening were investigated. The bacteria were identified by Phoenix 100 system. The clonal relatedness was evaluated by PFGE and semi-automated repetitive sequence-based PCR. Genotyping tests were repeated for 10 serial subcultures.

**Results:**

PFGE and DiversiLab yielded 10 genotypic profiles for 12 isolates. Four to eight different genotypes were observed from 10 subcultures of the same isolate.

**Conclusion:**

We conclude that, high genetic diversity and supposed multiclonal appearance of the outbreak isolates may be due to changing profiles during subcultures most probably depending on hypermutation.

## Introduction

*Stenotrophomonas maltophilia* is a non-fermentative gram-negative bacillus which is widely recognised as an important nosocomial pathogen causing pneumonia, blood-stream, wound and urinary tract infections, particularly in immunosuppressed patients. The bacterial spread is mostly via cross-transmission by contaminated equipment or environmental sources making an outbreak with this organism a significant challenge in intensive care units (1–3). Studies on properties of clinical *Stenotrophomonas* isolates frequently revealed a high genetic and phenotypic diversity (4–6). High genetic diversity of *S. maltophilia* isolates despite their origin from a single hospital irrespective of the time frame of collection in the same clinical setting was considered to be related to the wide environmental distribution of this pathogen (7). Some studies suggested that some phylogenetic groups may have increased potential to cause infections compared to others and some others demonstrated significant differences between mutation frequencies of environmental and clinical isolates, i.e., clinical isolates are more frequently hypermutators (8, 9). Molecular epidemiological studies including the detection of the microorganisms and related virulence properties, investigation of modes of transmission and the clonal relationship of the isolates are crucial for the determination of the source and implementing necessary control measures for prevention of outbreaks. Such molecular evaluation, together with the classical epidemiological data, leads to hypotheses for the prevention of outbreak transmission. However, thorough knowledge about the biological properties of the particular microorganism is required for an accurate interpretation of molecular studies. Unexpected results are reported in some molecular studies performed for *S. maltophilia* outbreak investigations emphasising the need for profound analysis of the epidemiological relationship among different isolates (7). The aim of this study was to evaluate a nosocomial outbreak of by *S. maltophilia* which occurred in a limited time with a limited number of isolates in an intensive care unit of a tertiary hospital where *S. maltophilia* was normally rarely isolated. Upon observation of unexpected genotypic hypervariability of the isolates during the study, the analysis of genotypic profiles after repeated molecular testing for 10 serial subcultures was also aimed.

## Material and Methods

### Study Design

Molecular epidemiological investigation of the nosocomial outbreak of *S. maltophilia* with repeated molecular testing.

### Patients and Bacterial Isolates

A total of 11 respiratory isolates from 11 patients in the 24-bed intensive care unit (ICU) of Near East University Hospital which is a tertiary 24-bed hospital were obtained in March 2015. Since this was the first isolation of *S. maltophilia* in the ICU of the hospital and the isolation rates increased significantly in a limited time, outbreak investigation including environmental sampling was done. A total of 35 samples were cultured from nebulizers, ventilators, bedsides, bed surfaces, floor surfaces and sinks during environmental screening. The isolates obtained from environmental samples were included in the study.

### Identification and Susceptibility Testing

The isolates were identified using Phoenix 100 automated system (Becton Dickinson, USA). Susceptibilities of the isolates to trimethoprim sulfamethoxazole, ceftazidime and levofloxacin were tested according to CLSI criteria (10).

### Molecular Epidemiological Studies

The clonal relatedness of the isolates was evaluated by pulsed-field gel electrophoresis (PFGE) and semi-automated repetitive sequence-based PCR (RepPCR) (DiversiLab typing). The molecular tests were repeated for 10 serial subcultures including the clinical and environmental isolates and *S. maltophilia* ATCC 17666.

### Pulsed–Field Gel Electrophoresis

PFGE analysis was performed as described by Shueh et al. with minor modifications. Isolates were digested with Xba restriction endonucleases (11). PFGE was performed using CHEF-DR II system (Bio-Rad Laboratories, Nazareth, Belgium) and run on a 1% agarose gel in 0.5x TBE Buffer at 14 °C with a linear ramp time of 5.3 s–52.4 s over a period of 24 h. The analysis of the PFGE banding profiles was done by GelCompar II system (version 3.0; Applied Maths, Sint-Martens-Latem, Belgium). The dendogram creation and cluster analysis was done by using Dice similarity coefficient and Unweighted pair group method with mathematical averaging (UPGMA). Isolates with Dice similarity coefficient ≥ 90% (tolerance of 1.5% in band position) were defined as “clonally related” (12).

### Repetitive Sequence-Based PCR

DiversiLab protocol was used following the manufacturer’s instructions. Isolates were cultured on blood agar for 24 h at 35 °C. Extraction of DNA was made with the UltraClean microbial DNA isolation kit (Mo Bio Laboratories, Inc., Carlsbad, CA) following the manufacturer’s instructions and extraction samples were diluted to 35 ng/μL. Repetitive sequence-based PCR (Rep-PCR) of extracted DNA was made using the DiversiLab Bacterial Kit (bioMérieux, Marcy l’Etoile, France). Briefly, 35 ng genomic DNA, 2.5 U AmpliTaq polymerase, 2.5 μL 10× PCR buffer (Applied Biosystems) and 2 μL primer mix were added to the Rep-PCR master mix in a total volume of 25 μL per reaction. Thermal cycling parameters were as follows: initial denaturation 94 °C for 2 min, followed by 35 cycles of denaturation at 92 °C for 30 s, annealing at 50 °C for 30 s and extension at 70 °C for 90 s, with a final extension at 70 °C for 3 min. Amplified fragments of various sizes and intensities were separated and detected with DNA chip (bioMérieux, Marcy l’Etoile, France) on Agilent 2100 Bioanalyzer (Agilent Technologies, Palo Alto, California). Results were demonstrated as dendrogram (with a Pearson correlation similarity matrix) including a virtual gel image of the fingerprint for each DNA sample. The similarity indexes defined for determination of clonal relationship between most gram-negative bacteria were used, i.e., isolates with 98% similarity of were considered indistinguishable (13).

### Plasmid Profile Analysis

Plasmid DNA was isolated by using Column-Pure Plasmid Mini-Prep Kit (Lamda Biotech, USA) following the manufacturer’s recommended protocol. The plasmid DNA restriction endonuclease patterns obtained by Bam H1, EcoRI, Hind III and Pst1 were analysed. The restriction enzymes were used in appropriate temperature and buffer conditions and gel electrophoresis with 0.8% agarose gel was done to visualisation the band fragments (14).

### Biofilm Production

Microtitre-plate assay and the quantitative analysis method were used for detection of biofilm production. Briefly, *S. maltophilia* cultures were inoculated in 3 mL tryptic soy broth (TSB; Oxoid, UK), and incubated in 37 °C for 24 h. The cultures were then diluted with 1:100 TSB, and 100 μL from each culture were placed into a well of 96-well microtiter plate, and the inoculated plates were incubated for 48 h at 37 °C. The wells were aspirated, washed with phosphate buffer saline (PBS, pH:7), stained with 0.1% crystal violet for 10 min, and then washed with tap water, and left to air dry. Following ethanol (95%) application, the contents were transferred to new wells, and the optical density (OD) of the each isolate was measured in a plate reader (Titertek Multiskan Plus, Flow Laboratories, Finland) at 630 nm wavelengths. The experiments were triplicate from the level of culture dilution with TSB, and the average and standard deviation values of OD were calculated for each strain. The strains harbouring an OD value of ≥ 0.5 were accepted as biofilm producer (15).

### Statistical Analysis

Software program SPSS version 3.0 (SPSS Inc. Chicago, IL, USA) was used for statistical analyses. Antibiotic susceptibility test results were analysed through ANOVA and tukeys HSD test. *P* < 0.05 was considered statistically significant (16).

## Results

### Patients and Isolates

In addition to 11 isolates obtained from clinical samples during the outbreak [S1–S11], one isolate was obtained from the nebuliser used in the ICU during environmental screening studies [S12]. All of the isolates were susceptible to trimethoprim sulfamethoxazole, ceftazidime and levofloxacin except for one isolate [S1] which was resistant to ceftazidime. The summary of patient data and antibiotic susceptibilities of the isolates are demonstrated in Table 1. There was a highly significant differences between the patients age and ceftazidime (*P* < 0.001); a significant differences between the sample type and PFGE (*P* > 0.95) and low significant differences between the sample type and DiversiLab (*P* < 0.001).

### Molecular Epidemiological Studies

#### Outbreak investigation

PFGE and DiversiLab yielded 10 genotypic profiles for 12 isolates. Two clinical isolates [S5 and S6] were demonstrated to be clonally related by both methods while all of the other isolates including the environmental isolate were unrelated. Banding patterns determined by PFGE and DiversiLab and the dendrograms showing the clonal relatedness of the isolates yielded a multiclonal outbreak as shown in Figure 1a–1b.

As the outbreak happened in a limited time with a limited number of isolates in a setting where *S. maltophilia* was rarely isolated and well-controlled with appropriate control measures, the unexpected multiclonality of the isolates were thoroughly investigated by repeated molecular testing.

### Repeated molecular testing of subcultures

**Figure 2:**
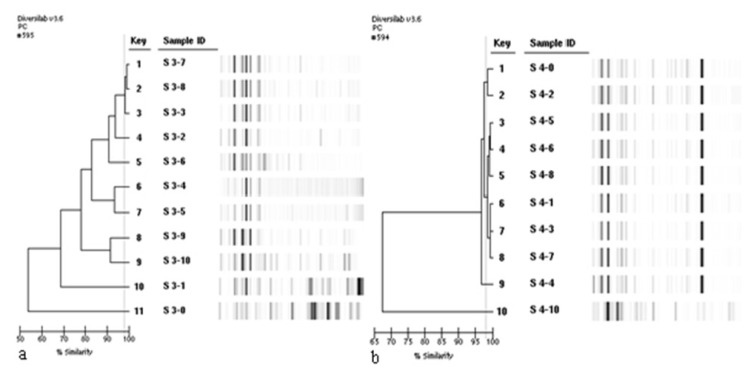
The alteration in the genotypic profiles of isolates S3 and S4 with serial subcultures obtained by DiversiLab system when 98% similarity threshold was used. a) Eight different genotypes from 10 subcultures were observed for isolate S3, b) Four different genotypes from 10 subcultures were observed for isolate S4

**Figure 3:**
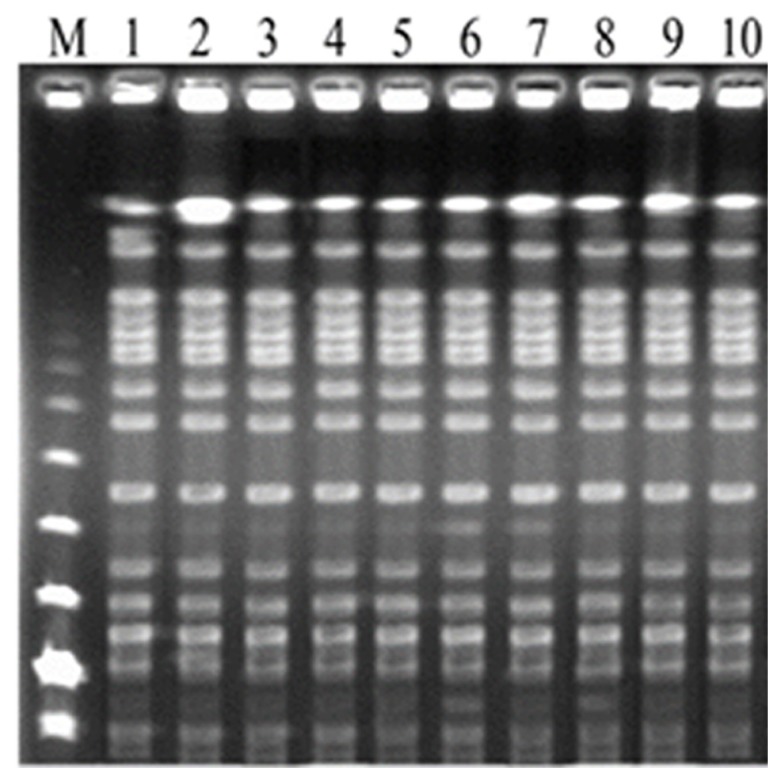
PFGE banding patterns of *S. maltophilia* ATCC 17666 after 10 subcultures. No alterations were observed after subcultures. M: PFGE Marker (Sigma)

The genotyping tests were repeated for all isolates including 10 serial subcultures of each isolate. PFGE and DiversiLab profiles of the clinical and environmental isolates changed significantly with subcultures while *S. maltophilia* ATCC 17666 remained to have similar patterns with repeated testing after subculturing. The alterations in the genotypic profiles with serial subcultures of two representative isolates and *S. maltophilia* ATCC 17666 are shown in Figure 2 and Figure 3. Four to eight different genotypes were observed from 10 subcultures of the same isolate.

### Plasmid profile analysis

**Figure 4:**
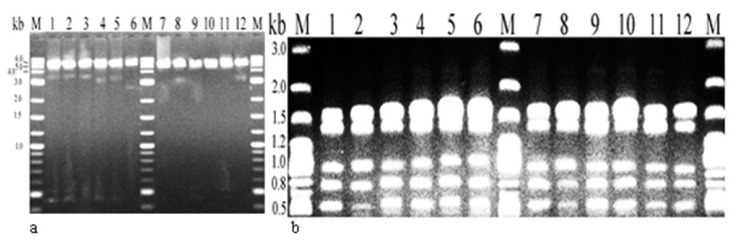
Plasmid profile analysis of the isolates. a) Plasmid DNA before endonuclease restriction, b) Plasmid profiles after endonuclease restriction (0.8% agarose gel was used for electrophoresis)

The plasmid DNA restriction endonuclease patterns obtained by Bam H1, EcoRI, Hind III ve Pst1 yielded the same plasmid profile for all of the isolates (Figure 4).

### Biofilm production

**Figure 5:**
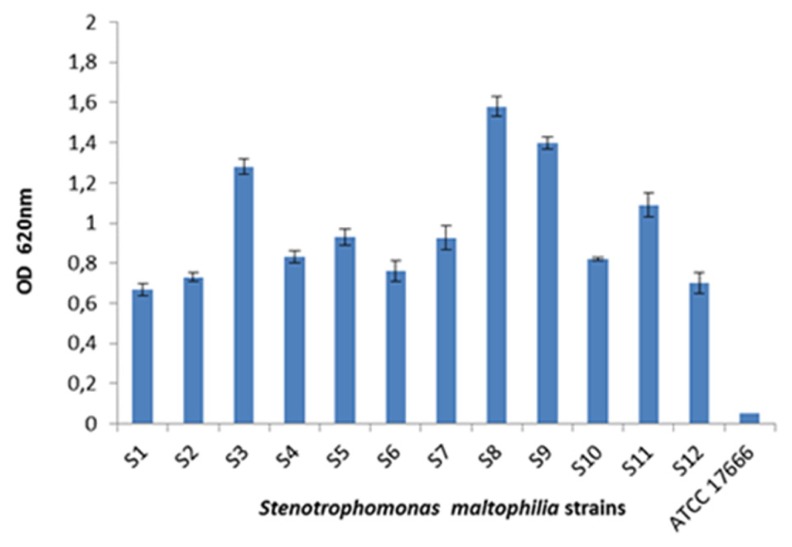
Graph showing different OD obtained for each isolate as calculated by the microtitre plate assay. The experiment was performed in triplicate form for each isolate. S1–S11; clinical isolates, S12; environmental isolate, S12, *S. maltophilia* ATCC 17666

Analysis of biofilm formation demonstrated that all clinical isolates and the environmental isolate were biofilm producers with OD values > 0.5 calculated by the microtitre plate assay while *S. maltophilia* ATCC 17666 did not produce biofilm (Figure 5).

## Discussion

Molecular epidemiological evaluation including genotyping methods is crucial for the definition, source-tracking and control of hospital outbreaks. Repetitive regions on the bacterial chromosome or restriction sites specific for particular restriction enzymes are frequently targeted for genotyping methods depending on DNA fragmentation (PFGE, Rep-PCR, AP-PCR, AFLP, etc.). These methods are based on the fact that the number and distance of the restriction sites/repetitive units of the isolates from the same origin will be similar and thus they will have the same/similar band profiles. The target regions used for the evaluation of clonal relations are usually selected incidentally in the aforementioned methods. However, the selected targets must be those with no mutation (cold regions) to be able to demonstrate strain variations. The incidental selection of the target regions in these methods brings with it the possibility to come across potential mutator regions depending on the properties of the particular microorganism/strain. A mutation in the bacterial chromosome may lead to alterations in the restriction sites and repetitive gene regions yielding changes in the DNA band profiles (17–18). Tenover et al. suggested criteria for the interpretation of PFGE result of isolates obtained in a particular setting and in a limited time. According to these criteria, a “mutational event” can occur during the transmission of the outbreak isolates from one patient to another, thus, isolates with one band difference can be considered same/indistinguishable; these isolates, which are considered genetically indifferent while they are epidemiologically related (18). Yet, one single mutation can alter the band profiles in five different ways, i.e., by changing the restriction sites with deletions and insertions (19). When such an alteration is investigated by using Dice similarity coefficient, which is frequently used for band fragment analysis, the similarity cut-off may decline up to 50% depending on the total number of bands. However, a similarity cut-off of 70%–80% is used for most of the bacteria and in some cases isolates from the same origin may appear different after one mutational event. For this reason, the genomic properties of the bacteria to be analysed by molecular epidemiological methods must be thoroughly known. Genotypic changes related to mutations, some of which were evidenced by sequence analysis, were demonstrated for several microorganisms with serial passages including *S. aureus*, *Trichophyton rubrum*, satellite tobacco mosaic virus (20–21).

Hypermutational strains are also reported among *S. maltophilia* isolates. Turrientes et al. demonstrated higher mutation rates for clinical *S. maltophilia* isolates when compared to environmental isolates (9). Besides, Valdezate et al. evaluated the clonal relatedness of 139 *S. maltophilia* isolates originating from a single hospital by PFGE and demonstrated high genetic diversity among the isolates; though they used similarity cut-off of 36% for their analysis of clonal relatedness, they obtained five large clusters (7).

In the present study, a limited number of *S. maltophilia* isolates with similar antibiotic susceptibility profiles were obtained in a limited time where *S. maltophilia* was normally rarely isolated and this was considered a nosocomial outbreak in the light of classical epidemiological data. The hospital infection control committee performed several meetings and screening cultures were taken yielding an environmental source, i.e., the nebuliser, for the outbreak. Education was given to the staff on sterilisation and disinfection procedures and preventive measures were taken leading to the control of the outbreak (no *S. maltophilia* was isolated from the ICU in the following two years). However, the molecular studies yielded high heterogenicity and multiclonality of the isolates which was considered unexpected. On the other hand, it was remarkable that all of the isolates carried a large single plasmid (approximately 5 Kb). The plasmid profile analysis showed that all of the isolates had the same plasmid profile which was taken into consideration as a significant evidence of probable clonal relatedness. Additionally, all of the clinical isolates as well as the isolate obtained from the nebuliser were high-level biofilm producers which was also considered a phenotypic marker of possible relation of the isolates. The molecular tests were repeated to exclude any laboratory failure to demonstrate the clonal relation and it was observed that banding patterns were different from the original isolates for most of the isolates. Repeated testing with 10 serial passages were done and profile changes were demonstrated from the very first subcultures of the isolates compared to the original isolates. We observed that the alterations in the percentages of similarity were incidental. We think that the intra-strain variability and PFGE profile changes in the present study may be due to hypermutation property of the outbreak isolates. Serial subcultures must be avoided before storage and advanced storage systems containing appropriate cryopresertavites with beads as microbanks must be preferred for long-term storage. Enrichment media and stressed growth conditions may have the potential to increase mutation rates and must be avoided during subcultures.

## Conclusion

High genetic diversity and supposed multiclonal appearance of the outbreak isolates may be due to profile changes during subcultures most probably depending on hypermutation. The clinical microbiologist must be aware of the particular genomic properties of outbreak isolates, particularly those with high potential of mutations like *S. maltophilia*, and be attentive during the interpretation of data obtained from molecular studies of high-passage isolates and make epidemiological conclusions with caution and in conjunction with genotyping and classical epidemiological data including traditional contact tracing information.

## Figures and Tables

**Figure 1a:**
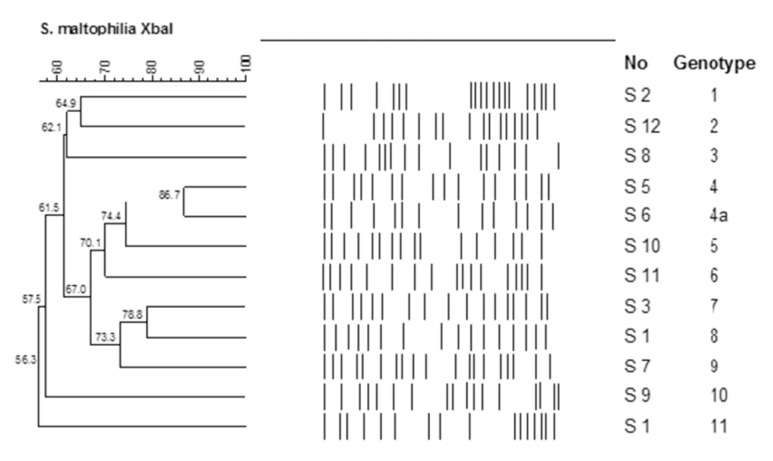
Dendrogram of PFGE patterns showing similarities of 11 clinical *S. maltophilia* isolates [S1–S11] and one isolate obtained from the nebuliser used in the ICU [S12]. Dice coefficient and the “unweighted pair-group method with arithmetic mean” (UPGMA) cluster method were used for the dendogram analysis

**Figure 1b:**
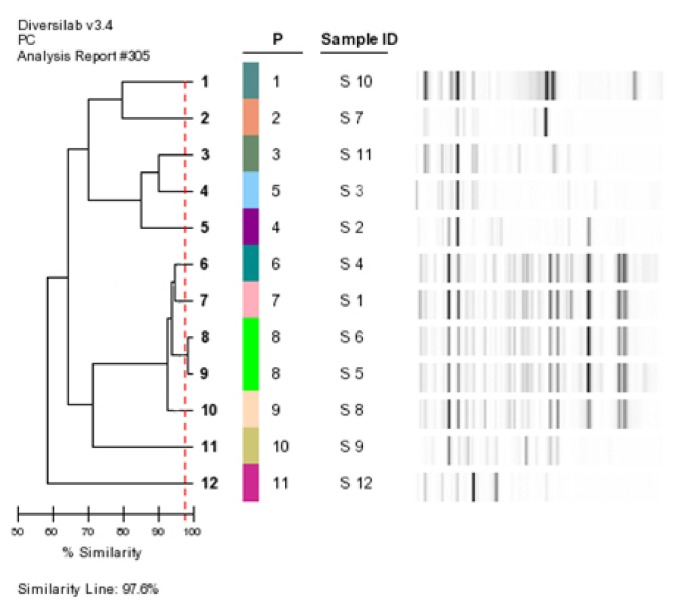
Rep-PCR-based dendrogram and virtual gel image fingerprints obtained from 11 clinical isolates [S1-S11] and one environmental isolate [S12] using the DiversiLab system. Pearson’s correlation coefficient was used to create a pairwise percentage similarity matrix and the dendogram was derived using UPGMA. An 98% similarity threshold (vertical line) was chosen for *S. maltophilia*

**Table 1:** The summary of patient data and characteristics of the isolates

Strain No	Gender	Age	Sample type	Isolation date	Hospitalised for	Intubation	Antimicrobial susceptibility			Typing		
							CAZ	LEV	SXT	PFGE	DL	PPA
S1	M	40	DTA	01.03	CVA	+	>16 (R)	≤1 (S)	≤1 1/19 (S)	11	7	A
S2	M	35	Sputum	02.03	CVA	+	4 (S)	≤1 (S)	≤1 1/19 (S)	1	4	A
S3	M	42	Sputum	02.03	CVA	+	4 (S)	2 (S)	≤1 1/19 (S)	7	5	A
S4	M	50	DTA	04.03	MI	+	4 (S)	≤1 (S)	2/38 (S)	8	6	A
S5	M	65	DTA	12.03	CVA	−	4 (S)	≤1 (S)	≤1 1/19 (S)	4	8	A
S6	M	60	DTA	12.03	CVA	+	4 (S)	≤1 (S)	≤1 1/19 (S)	4a	8	A
S7	M	70	Sputum	14.03	MI	+	4 (S)	≤1 (S)	≤1 1/19 (S)	9	2	A
S8	F	65	Sputum	15.03	ICH	+	4 (S)	≤1 (S)	≤1 1/19 (S)	3	9	A
S9	F	62	DTA	17.03	CVA	+	4 (S)	≤1 (S)	≤1 1/19 (S)	10	10	A
S10	F	60	Sputum	21.03	CVA	+	4 (S)	≤1 (S)	≤1 1/19 (S)	5	1	A
S11	M	72	Sputum	25.03	CVA	+	4 (S)	≤1 (S)	≤1 1/19 (S)	6	3	A
S12			Nebuliser	20.03			4 (S)	≤1 (S)	≤1 1/19 (S)	2	11	A

MI; Mitral insufficiency, CVA; cerebrovascular attack, ICH; Intracranial hemorrhage,

CAZ; ceftazidime, LEV; levofloxacin, SXT; trimethoprim-sulfamethoxazole
